# Challenges in HIV Diagnosis Algorithm: Experience of the Confirmation Laboratory

**DOI:** 10.1155/jotm/5111633

**Published:** 2025-01-31

**Authors:** Özgür Appak, Derya Özarslan, Arzu Nazlı, Ayca Arzu Sayiner

**Affiliations:** ^1^Faculty of Medicine, Department of Medical Microbiology, Division of Medical Virology, Dokuz Eylul University, Izmir, Turkey; ^2^Faculty of Medicine, Department of Medical Microbiology, Dokuz Eylul University, Izmir, Turkey; ^3^Faculty of Medicine, Department of Infectious Disease and Clinical Microbiology, Dokuz Eylul University, Izmir, Turkey

**Keywords:** algorithms, confirmatory, diagnosis, HIV

## Abstract

This study aimed to evaluate the effectiveness of the algorithm used in HIV diagnosis and to propose an effective new algorithm for rapid diagnosis. In accordance with CDC algorithm, our laboratory uses Architect HIVAg/Ab for screening and Geenius HIV1/2 and Artus HIVirus-1 QS-RGQ for confirmation. The Geenius test was used as a reflex and the HIV-1-RNA required clinician order. The HIVAg/Ab test was performed in 82,882 sera and found to be reactive in 262 (0.3%). HIV-antibody confirmatory testing was performed on 79% of samples with a reactive screening test, and the presence of HIV-1 antibodies was confirmed in 51% (105/206). Half of the samples with positive-screening but negative-antibody confirmatory results were tested for HIV1-RNA, and viremia was detected in 5, confirming acute HIV1 infection. HIV1-RNA was not ordered for 49 samples with positive-screening and negative antibody-confirmation tests, and 16 of these were considered false-reactive by the clinician. The Geenius assay result was indeterminate in 1.45% (3/206) of the samples. In the algorithm, the number of Geenius tests would have been reduced by 25% if HIV-1-RNA had been applied as a reflex test to HIV-Ag/Ab positive samples and Geenius testing had been performed on RNA negative samples. A retrospective analysis showed that the HIV diagnostic algorithm was not fully implemented. An important factor was that clinicians did not order HIV-1-RNA-PCR from ELISA reactive and Geenius test negative patients. Requesting HIV-1 RNA PCR as a reflex test is thought to prevent patient losses and shorten the turnaround time of the HIV diagnosis.

## 1. Introduction

Approximately 1.5 million people worldwide are diagnosed with human immunodeficiency virus (HIV) infection every year. While the availability of antiretroviral therapy (ART) dramatically enhances the life quality and longevity of individuals with HIV, it is notable that in 2021, an estimated 650,000 people globally succumbed to AIDS-related diseases [1, 2]. The Joint United Nations Programme on HIV/AIDS (UNAIDS) aims to end the HIV epidemic by setting ambitious 95-95-95 targets in response to the associated morbidity and mortality. These goals state that by 2030, 95% of all people living with HIV will know their status, 95% will consistently be on ART, and 95% undergoing ART will achieve viral suppression [3].

There are many benefits to early detection of HIV infection. Starting treatment earlier reduces the risk of developing serious events associated with AIDS and death by at least 50% [4, 5]. Additionally, several studies have shown that starting treatment within 1 week to 1 month after starting ARVs slows the progression of the disease, improves the immune system, and reduces the risk of viral reservoir and treatment failure [6–9]. Moreover, treatment also prevents the transmission of HIV to other people. In its revised 2017 HIV care and treatment guidelines, the World Health Organization (WHO) recommends that ART be initiated quickly, within seven days of a positive HIV diagnosis [10].

Several tests are used in the diagnosis of HIV. However, no test can detect the virus immediately after infection. Currently, antibody tests, antigen/antibody tests, and nucleic acid tests (NATs) are used for diagnosis. Typically, after exposure, antibody tests can detect HIV between 23 and 90 days, antigen/antibody tests between 18 and 45 days, and NAT within 10–33 days. NAT should be considered for individuals who have recently been exposed or have had a possible exposure with early symptoms of HIV and tested negative with antibody or antigen/antibody tests [11].

In order to accelerate the diagnosis and reporting process in the control of the HIV epidemic and to start treatment as soon as possible, the Centers for Disease Control and Prevention (CDC) in the United States recommended the use of rapid verification tests for confirming positive screening tests and published a new diagnostic test algorithm in 2010 [12]. This algorithm, which was first published in 2011 by the Clinical Laboratory Standards Institute (CLSI) and later in 2014 in the CDC's diagnostic guide, aimed to facilitate early initiation of treatment [13]. In our country, the transition process to this algorithm was completed in August 2019 and published by the Ministry of Health under the name “HIV-AIDS Diagnosis and Treatment Guide” [14].

Our study aims to share the issues and experiences at the HIV verification center between January 2020 and December 2023, evaluate the effectiveness of the employed algorithm, and suggest a new, efficient algorithm for rapid diagnosis.

## 2. Materials and Methods

Our study included the results of pediatric and adult patients of all age groups who sent samples for HIV diagnosis to Dokuz Eylul University Central Laboratory between January 2020 and December 2023. HIV Ag/Ab, HIV 1/2 confirmation, and HIV 1 RNA PCR results of the patients were obtained retrospectively through the Laboratory Information System (LIS).

HIV diagnosis at DEU Central Laboratory was conducted using Architect HIV Ag/Ab Combo assay kits (Abbott Laboratories, Chicago, IL) from January 2020 to June 2022 and Alinity i HIV Ag/Ab Combo kits (Abbott Laboratories, Chicago, IL) from July 2022 to December 2023, while the Geenius HIV 1/2 confirmatory test (Bio-Rad Laboratories, Redmond, WA) and Artus HI Virus-1 QS-RGQ kits (QIAGEN, Germany) were utilized throughout the study period.

### 2.1. Architect HIV Ag/Ab Combo Assay and Alinity-i HIV Ag/Ab Combo Assay (Abbott Laboratories, Chicago, IL)

During different periods of our work, commercial kits manufactured by Abbott Company using chemiluminescent microparticle enzyme immunological (CMIA) technology were used for HIV Ag/Ab testing. These kits qualitatively detect p24 antigen and anti-HIV 1/2 antibodies in human serum/plasma, and they were operated on Architect i2000 and Alinity-i analyzer systems. According to the test procedure, samples that have an S/Co value of less than 0.90 for HIV Ag/Ab are deemed negative, those between 0.9–0.99 are viewed as uncertain, and those with a value of 1.00 or greater are regarded as positive.

### 2.2. Geenius HIV 1/2 Confirmation Test (Bio-Rad Laboratories, Redmond, WA)

In instances of repeated reactivity detected via the ELISA test, we used the immunochromatographic-based Geenius HIV 1/2 confirmation test (Bio-Rad Laboratories, Redmond, WA) to confirm the presence of antibodies developed against HIV-1/2 and identify the virus type. This test is performed in our laboratory as a reflex test, irrespective of clinicians' requests. The test comprises four HIV-1 antigen (p31, gp160, p24, and gp41) and two HIV-2 antigen (gp36 and gp140) bands. The test was conducted according to the manufacturer's instructions, and after 30 min, the test cassettes with a positive control line were visually evaluated. For interpretation as HIV-1 positive, at least two out of four HIV-1 bands must be positive, with at least one belonging to envelope antigens. For HIV-2, positivity requires both gp36 and gp140 bands to be positive together. If bands show positivity other than the envelope proteins of HIV-1, or only one band is positive, or if only one envelope protein of HIV-2 is positive, the test is reported as indeterminate.

### 2.3. Artus HIV-1 QS-RGQ Kit (QIAGEN, Germany)

The Artus HI Virus-1 QS-RGQ kit (QIAGEN, Germany), which operates on the principle of real-time RT-qPCR, was utilized to quantitatively detect the presence of HIV-1 RNA in plasma. The target is a 93 bp region in the 5′UTR region of the HIV-1 genome. The manufacturer reports that the analytical sensitivity of the HIV-1 RNA PCR test is 34.4 copies/mL (1IU = 0.45 copies) using the International WHO Standard dilution series (NIBSC Code 97/650), with a quantification range of 45–4.5 × 107 copies/mL. Nucleic acid extraction was performed using the QIAsymphony SP instrument with the QIAsymphony virus kit (QIAGEN).

### 2.4. Ethics Committee

Our study was carried out with the approval of the DEU Non-Interventional Research Ethics Committee (03.01.2024, decision no: 2024/01–25).

## 3. Results

**Figure 1:**
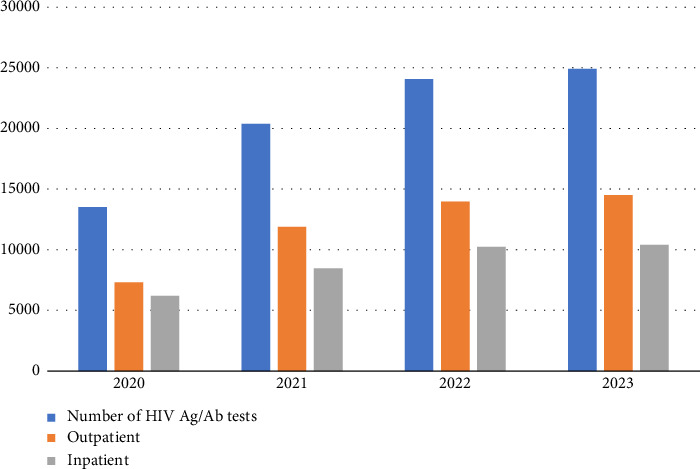
Number of HIV Ag/Ab tests according to the years.

In our study, HIV Ag/Ab testing was performed on 82,882 patients between the years 2020 and 2023 (Figure 1), and the results were found to be reactive in 0.3% (262/82,882) of patients. The reactivity rates by year were 16% (42/262), 25.2% (66/262), 32% (84/262), and 26.7% (70/262), respectively. The average age of patients was 41 years (min 4-max 89), with 71% (185/262) being male and 29% (77/262) female. Of the reactive samples, 73% (190/262) were from outpatients, and 27% (72/262) were from inpatients. The percentage of HIV patients newly diagnosed among the reactive patients was 45.2% (19/42), 33.3% (22/66), 38.1% (32/84), and 54.3% (38/70) by year, respectively.

**Figure 2:**
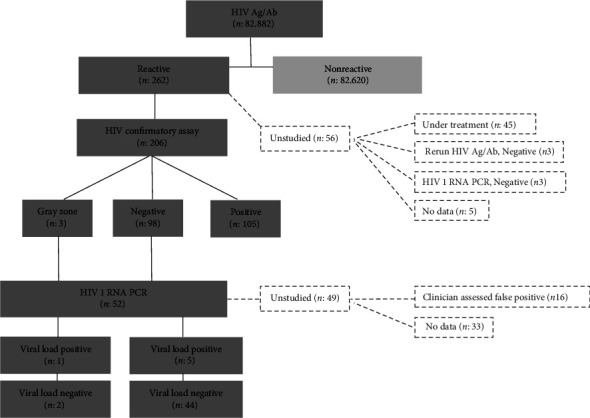
Data on the application of the CDC HIV diagnosis algorithm in our center.

Of the patients with reactive HIV Ag/Ab results, HIV antibody confirmation testing was conducted for 79% (206/262) of them. Of these, 51% (105/206) were found to have a positive result for the HIV-1 antibody (Figure 2). Among the 56 patients who were not confirmed, 80.4% (45/56) were either from outside facilities or previously diagnosed patients. Among the remaining 11 patients, 3 had a nonreactive HIV Ag/Ab test repeated 2–3 weeks later, and the other 3 had negative results for HIV-1 RNA PCR test. For the 5 patients for whom ELISA repeat, confirmation, and HIV-1 RNA PCR testing were not conducted, the average HIV Ag/Ab S/Co value was 1.63 (1.26–2.31). Three of these five patients were among those for whom HIV Ag/Ab was requested for preoperative preparation. One patient was found to have a negative HIV-1 RNA PCR result outside our study period (02/01/2024). The other patient was a 70-year-old man with multiple sclerosis. He had no history of HIV risk contact.

Of the patients whose HIV-1 antibody confirmation result was negative, making up 37.4% (98/262), HIV-1 RNA PCR was performed on 50% (49/98) of these patients. Among these, acute HIV infection was considered in 5 patients where viral load (13,621 copies/mL–19,498,277 copies/mL) was detected, and a false HIV Ag/Ab reaction was considered in 44 patients where HIV-1 RNA PCR was negative (Figure 2). The average S/Co values of these patients were 9.73 (1.09–96.52). Among the 49 patients who were not tested with HIV-1 RNA PCR, 14 were considered falsely reactive based on information obtained from clinicians. Two pediatric patients were referred to an outside center for follow-up, one patient did not provide the required blood for HIV-1 RNA PCR, and three patients identified in blood center screening failed to follow up at the hospital. There was no information available for the remaining 29 patients in the records. The average S/Co values of these patients were 3.06 (1.02–22.09). Of the patients who did not request HIV-1 RNA PCR, 45% (13/29) were among those screened for preoperative preparation. Of the remaining patients, 7 were inpatients and 9 were outpatients. The mean age of the inpatients was 70.2 years (min 48-max 88), and they had hypertension, coronary artery disease, congestive heart failure, colitis, cholangitis, familial Mediterranean fever, and depression. 7 of 9 outpatients had hematologic (breast cancer, Hodgkin's lymphoma) and rheumatologic (Sjögren's syndrome, rheumatoid arthritis) diseases. These patients had no history of risk factors for HIV. Of the remaining outpatients (2/9), one was a 29-year-old male patient with a history of suspected sexual intercourse, anti-*Treponema pallidum* IgM, IgG reactive (S/Co: 22.78), and rapid plasma reagin 1/32 positive, and the other was a 33-year-old male patient with diarrhea complaints.

In our study, we determined the confirmation result as uncertain at a rate of 1.45% (3/206). Among these 3 patients, isolated band 2 positivity was detected in 2 patients, and isolated band 6 positivity was detected in the other patient. The HIV-1 RNA PCR results of patients with isolated band 2 positivity and HIV 2 RNA PCR results from the reference laboratory were determined to be negative. The HIV-1 RNA PCR result of the patient with isolated band 6 positivity was determined as 180,265 copies/mL.

## 4. Discussion

Early diagnosis is crucial to preventing HIV transmission and achieving effective treatment. In our laboratory, the CDC's HIV diagnostic algorithm is used for this purpose. This algorithm recommends that reactive results after screening with a fourth-generation ELISA test be confirmed with HIV-1/2 antibody differentiation rapid verification test, and that confirmed negative or indeterminate results be tested with HIV NATs to rule out acute HIV infection [15].

**Figure 3:**
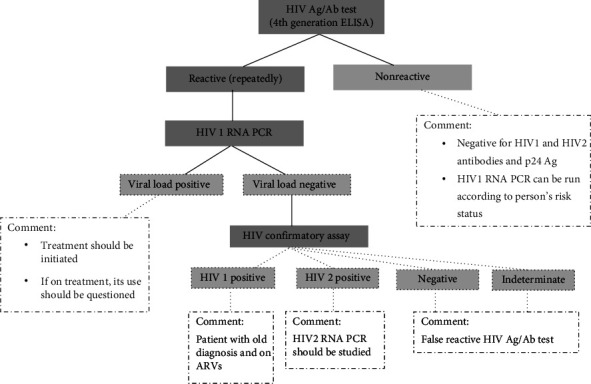
Schematic view of an alternative HIV diagnostic algorithm.

In our study, HIV Ag/Ab test was performed on 79% of patients with a reactive result (206/262) for the HIV 1/2 confirmation test. The 56 patients who were not tested in the confirmation step included those with a prior diagnosis, those who repeatedly tested nonreactive to the HIV Ag/Ab test, and those with a negative HIV-1 RNA PCR result. Only 5 patients were identified who did not undergo confirmation and/or HIV-1 RNA PCR tests. It was found that 50% of patients with negative or indeterminate confirmation results did not have HIV-1 RNA PCR tests requested. In a retrospective study similar to ours, 35% of individuals with discordant results on HIV Ag-Ab screening immunoassay and antibody differentiation testing did not undergo confirmatory HIV NAT, and of those who did, 12% had a positive result. They concluded that an institutional testing algorithm without an HIV NAT reflex misses opportunities for confirmatory testing [16]. Another study evaluating the performance of the current HIV testing algorithm found that 22% of patients with a positive screening test and a negative confirmatory test did not provide a specimen for HIV NAT [17]. In our study, two patients with incompatible screening and confirmatory tests (newly diagnosed syphilis and diarrhea patients) can be considered as missed diagnoses because their algorithms were not completed. It should be noted that such cases may be missed if the current algorithm is not completed. In our laboratory, ELISA reactive and confirmation positive results are deemed critical (panic) values for HIV assessment. We communicate the patient results to clinicians via phone and advise them to request an HIV-1 RNA PCR test. Plasma samples collected in EDTA tubes were used for HIV-1 RNA PCR in our study. It is believed that collecting a second tube of blood from the patient may hinder the completion of the algorithm's third stage. In a study conducted by Kaperak et al., routine HIV screening program data were examined before and after the implementation of reflex HIV-1 RNA testing. The results indicated that reflex testing aided in the confirmation of reactive HIV screening tests (true or false positive) (odds ratio, 23.7 (95% confidence interval, 6.7–83.4); *p* < 0.0001), enhanced acute HIV detection, and decreased unconfirmed discordant results. Furthermore, we prioritized contacting patients with true positive results, rather than following up on individuals with discordant results [18]. Pitasi et al. conducted a study to evaluate the performance of quantitative HIV-1 RNA viral load testing following HIV Ag/Ab testing and compared five different Ag/Ab tests in the process. They found that the sensitivities of Ag/Ab tests are high in established infection (≥ 99.4%), but low in acute infection (31%–54%), and all tests had similarly high specificities. They highlighted that two distinct commercial kits for HIV viral load assessment in their proposed methodology produced similar outcomes. They also reported that the application of viral load testing for both diagnosis and prognosis could minimize the total number of tests required [19]. In our study, the S/Co values of 44 samples evaluated as false reactive were 9.73 (1.09–96.52). Due to the high sensitivity of the 4th generation HIV Ag/Ab tests used as screening tests, false positivity is frequently detected in low prevalence populations. Therefore, diagnosis is supported by confirmation tests. Studies suggesting that S/Co values can be used to predict HIV diagnosis have reported different ratios such as 26.25 S/Co, 3.78 S/Co, 8.82 S/Co, and 13.16 S/Co [20–23]. However, factors like HIV prevalence, test method, and patient population influence these cutoff values. In our study, the confirmation and HIV-1 RNA PCR results of 3 patients with S/Co values of 44.69, 68.07 and 96.52 were found to be negative. Therefore, assessing patients as positive or negative based solely on cutoff values is often deemed incorrect. In our study, the mean S/Co values of patients who did not undergo HIV-1 RNA PCR testing but had negative confirmation results were 3.06 (1.02–22.09), which is thought to lead clinicians to misinterpret the results as falsely reactive. Conducting HIV-1 RNA PCR at the second stage instead of confirmation may help prevent patient dropouts and allow for earlier diagnosis of acute infection, thereby permitting early treatment (Figure 3). The Geenius HIV 1/2 confirmatory test and Artus HIV-1 QS-RGQ kit used at our center cost $71 and $30 per patient, respectively. Our proposed algorithm requires specialized personnel and laboratory infrastructure for PCR testing. Although this increases the cost, we believe that fewer patients will require HIV 1/2 differential confirmatory testing and the turnaround time will be shorter. In fact, in terms of test cost per patient, using the algorithm will be less expensive for our center.

In our study evaluating the CDC algorithm, the mean reporting time for confirmatory testing was found to be 3 h and 50 min after ELISA testing. The mean reporting time for HIV-1 RNA PCR was 12.79 days (±10.22) after ELISA testing and 12.63 (±10.12) days after confirmatory testing [23]. With the HIV algorithm we propose, this time will be reduced to 1-2 days at our center. Nowadays, the increasing use of ARTs before and after HIV exposure can alter virological and immunological responses. Suppression of HIV viral replication can lead to a decrease in viral load and delay in seroconversion. Therefore, it should not be forgotten that early treatment can alter the results of commonly used screening and confirmation tests [24]. Therefore, it is important for HIV test requests to be made as consultations from laboratories, sharing patient history and information for the evaluation of test results.

Our country lacks an approved laboratory test for HIV2 RNA. Blood samples from suspected HIV2 cases are sent to the Ministry of Health's National Virology Reference Laboratory, where they are analyzed by in-house HIV2 RNA PCR. Since HIV2 cases are sporadically reported, we plan to start with HIV-1 RNA PCR test in the second stage of our algorithm; in the event of negative results, we plan to work on HIV 2 RNA according to the Geenius differential test result. However, Geenius shows strong performance for HIV-1 tests when visual reading is used, while its discriminatory performance for HIV-2 is less reliable. Since HIV-2 infection has been reported sporadically in Japan, the use of Geenius Reader is recommended to differentiate between HIV-1 and HIV-2 [25]. It is therefore necessary to use a reader in our center to avoid missing HIV2 cases.

The important limitations of our study include the retrospective nature of our work, the recommended algorithm not being applied in real time, and a comparison study not being conducted on the costs of Geenius and RNA PCR tests.

In conclusion, our study has shown that the HIV diagnostic algorithm was not fully implemented. An important factor was that clinicians did not request HIV1-RNA-PCR from ELISA reactive and Geenius test negative patients. It is expected that using HIV1-RNA-PCR as a reflex test in HIV-Ag/Ab reactive samples will provide earlier diagnosis and reduce the use of Geenius testing, resulting in less uncertain results and decreased diagnostic costs.

## Data Availability

The data that support the findings of this study are available on request from the corresponding author. The data are not publicly available due to privacy or ethical restrictions.
